# A simulation experiment study to examine the effects of noise on miners’ safety behavior in underground coal mines

**DOI:** 10.1186/s12889-021-10354-2

**Published:** 2021-02-09

**Authors:** Jing Li, Yaru Qin, Lei Yang, Zhen Wang, Ke Han, Cheng Guan

**Affiliations:** College of emergency management and safety engineering, China University of Mining and Technology, Beijing, China

**Keywords:** Coal mine noise, Safety behavior ability, Attention distribution, Reaction, Fatigue

## Abstract

**Background:**

Noise pollution in coal mines is of great concern. Personal injuries directly or indirectly related to noise occur from time to time. Its effects impact the health and safety of coal mine workers. This study aimed to identify if and how the level of noise impacts miners’ safety behavior in underground coal mines.

**Methods:**

In order to study the influence of noise on miners in the mining industry, we built a coal mine noise simulation experiment system, and set the noise test level at 50 dB ~ 120 dB according to the actual working environment at well. We divided the noise gradient into 8 categories and conducted 93 experiments, in which we aim to test miners’ attention distribution, fatigue, and reaction under each level, and the experimental results were analyzed by SPSS22.0 software.

**Results:**

The results show that the increase of environmental noise level will have an impact on the attention, reaction, and fatigue. The noise is positively related to the fatigue, the noise is negatively related to the attention and reaction. In the noise environment, the sensitivity of the personnel to optic stimuli is higher than that to acoustic stimuli. The test indicators of attention, fatigue, and reaction will change significantly, when the noise level is greater than 70 ~ 80 dB.

**Conclusions:**

From the perspective of accident prevention, the noise level can be controlled within the range of less than 70 ~ 80 dB, which can control the occurrence of accidents to a certain extent.

## Background

Coal mine production accidents occur frequently to China. Accident statistical analysis shows that 96.5% coal mine accidents were caused by human errors [1]. Coal mine noise is one of the important causes of human errors. It comes from industrial equipment in production activities [2], such as shearers, tunneling machines, ventilators, conveyors, rock drills, pneumatic drills, etc. [3] Also, noise from underground coal mine has multiple sound sources, high intensity, high sound level, and frequency bandwidth [4]. Relevant data [3, 4] and previous field research findings revealed that the noise level in most underground mine operating environment has reached more than 90 dB. This figure exceeds 85 dB (Work 5d a week, 8 h a day, the steady-state noise limit is 85 dB (A)), the maximum health limit in the “*Occupational Exposure Limits for Hazardous Agents in The Workplace - Physical Factors” (GBZ2.2–2007)* [5]. Coal mine noise negative impacts on miners’ psychology, physiology, and behavior, affecting coal mine safety production [4, 6, 7].

Psychologically, noise impacts people’s mood and is likely to produce irritability [8].

Physiologically, scholars have conducted a lot of researches on how noise affects hearing, heart rate, and blood pressure. Noise affects human auditory organs, nervous system, and cardiovascular system, etc. [9–14]. Basner et al. [15] studied the effects of noise on hearing, and found hearing loss caused by noise is very common in working environments. Studies [16–22] have also found that high noise levels can cause hearing loss and general health problems. Masterson et al. [23] studied the hearing loss of workers exposed to noise from 2003 to 2012 and found 76% mining workers are exposed to dangerous noise. It was the highest among all industries. They suffered most from hearing impairment among all industries. Early researches [24, 25] found blood pressure and heart rate increase in long-term exposure to noise. Tian et al. [26] found subjects’ heart rate would increase in noise environment. Scholars have also explored the relationship between noise and blood pressure, and their conclusions varied about whether and how noise affects blood pressure. Hessel et al. [27] found that occupational noise exposure had no effect on blood pressure. However, Liu et al. [28] suggests that noise in working environment contributes to hypertension and can increase systolic and diastolic blood pressure.

Coal mine noise affects the safety behavior of miners and causes safety accidents. Current researches show that noise in the workplace has a significant impact on the behavior of workers [4, 29–32]. Behavior refers to the physical, psychological, and action responses to external stimuli. As an external stimulus, noise changes people’s physiology, psychology, and actions, and affects people’s behavior. Cheng et al. [6] studied how coal mine noise affects physiology and psychology of miners, and the impact of noise on human safety behavior. They found that noise has a serious negative effect on the miners’ safety behavior. Deng [33] states that noise has an impact on physiology and psychology, and then affects human behavior and leads to safety accidents. Yu et al. [34] compared the accidents in two factories, and found accidents in a 95 dB are significantly more than in 80 dB; accidents in a noisy environment is 20 times higher than in a quiet environment. Wang et al. [35] studied how noise influences miners’ behavior ability, and found the behavior ability of miners in a strong noise environment of 85 dB and 95 dB is significantly lower than in lower noise environments. Some of these studies found that [36, 37] noise impacts human attention, and noise above 85 dB would have a greater negative impact on human attention. Reaction time would prolong under strong noise [38]. Tian et al. [29] compared two groups of workers. One group has more knowledge and better awareness about safety production than the other group. The study found that noise has a greater impact on miners with a lower level of knowledge and awareness about safety production.

In general, attention, reaction, and fatigue are three most often studied behavioral ability indicators. Attention means the ability to focus. Attention distribution ability is how accurate to conduct multiple tasks at the same time. In other words, it means how well one can pay attention to different objects at the same time. When workers operate the equipment continuously for a long time, they are often fatigue and their working efficiency decrease [39]. Reaction ability means the response to stimuli signals. First, stimuli are felt by nerve system. Then, it is transmitted from the nerve system to the brain. Brain processes the stimuli and produces instructions to the muscles via nerve system and directs muscle contraction. Reaction ability is evaluated by the reaction time to the stimuli, the time duration from the moment when the external stimulus is received by the nerve system to the completion of reactive behavior by muscles [31]. In addition, noise is commonly believed to be positively correlated with fatigue [40]. Fatigue is often measured by the flicker fusion frequency. And the lower the flash fusion frequency is, the more fatigue the human body are [40]. When being fatigue, people will slow down their thinking and movements, lose concentration. In this case, coordination and accuracy of movements decline and safety behavior ability reduces [41].

The above researches on the impact of noise on people mainly focus on occupational hazards, and relatively little researches has been conducted on the effects of noise on human behavior. However, most of the above studies regard noise as an overall influencing factor, and they haven’t divided noise into different levels. Very few of them studied on simulation experiments of real noise environment in coal mines. Therefore, we aimed to explore the relationship between noise changes and miners’ behavior ability changes. To do this, this paper built an independent coal mine noise simulation experiment system, and divided the noise data collected in the real coal mine into 8 levels, and studied how 8 noise levels influence the safety behavior of miners in terms of attention, reaction, and fatigue. We hope this study could provide new ideas for underground coal mine noise prevention and coal mine accident prevention.

## Methods

In this quantitative research, attention, reaction, and fatigue are selected as research indicators after referring to the relevant literature [26, 42, 43].

### Experimental system design

The experimental system consists of a noise control system and a safety behavior ability testing system.

The noise control system consists of noise source, a louder speaker box, a sound meter, and a computer. The noise from the underground coal mine is collected as the noise source. The noise levels are precisely controlled through the louder speaker box and the sound meter.

The safety behavior ability testing system consists of an attention distribution meter, a multiple reaction meter, and a flicker frequency fusion meter. They test the changes in attention, reaction, and fatigue level on 8 noise levels. The specific description is as follows.

#### Attention

The experiment selected BD-II-314 attention distribution meter, to measure the subjects’ attention distribution. The meter tested subjects’ ability to perform two tasks at the same time. Q value of attention distribution was used to indicate attention distribution. It is calculated by Eq. 1:

1 $$ Q=\sqrt{\left({S}_2/{S}_1\right)\times \left({F}_2/{F}_1\right)} $$

Note: *S*_*1*_ indicates the times of correct reactions to acoustic stimuli; *S*_*2*_ indicates the total times of reactions to the acoustic stimuli; *F*_*1*_ indicates the times of correct reactions to optic stimuli; *F*_*2*_ indicates the total times of reactions to optic stimuli.

The meaning of Q value is as follows:

- When Q < 0.5, there is no attention distribution value;
- When 0.5 ≤ Q < 1.0, only a part of the total attention is assigned;
- When Q = 1.0, the attention distribution value reaches the highest level. It means the efficiency of performing multiple tasks simultaneously is equal to the efficiency when doing a single task.
- When Q > 1.0, the attention distribution value is invalid.

#### Reaction ability

In our study, we used BD-II-509B multiple reaction time tester to measure the reaction ability of the subjects to acousto-optic stimuli.

#### Fatigue

This study used the BD-II-118 flicker frequency fusion meter to measure the critical flicker fusion frequency of the subjects.

The noise test equipment is shown in Table 1.

**Table 1:** Noise test equipment

Experiment System	Device Name	Equipment Model	Manufacturer	Certification Information
Noise Control System	Computer	Dell- inspiron 7557		
	Louder Speaker Box	JBL- Charge4		
	Sound Meter	TES1350A		
Safety Working Ability Testing System	Attention Distribution Meter	BD- II-314	Beijing Qingniao Tianqiao Instrument Equipment Co., Ltd.	ISO9001: 2015 quality management system certification
	Multiple Reaction Time Tester	BD- II-509B		
	Flicker Frequency Fusion Meter	BD- II-118		

There are 8 noise levels in our tests, one control group and seven experimental groups. The noise level of control group was 50 dB. The seven experimental groups were 60 dB group, 70 dB group, 80 dB group, 90 dB group, 100 dB group, 110 dB group, and 120 dB group. This is because through reviewing the literature and on-site investigation, we found the range of underground coal mine noise is mainly between 90 dB and 120 dB [4, 6]. Also, this experiment also set 4 noise levels below 90 dB to explore the influence of a wider range of noise on workers’ safety behavior ability in order to improve the credibility of the experimental results.

The subjects of this study are healthy male graduate and undergraduate students aged from 20 to 25. During the experiment, the subjects did not use any personal protective equipment. In the early stage of the experiment, 14 subjects were selected to conduct experimental tests with 5 noise levels (50 dB,60 dB,70 dB,80 dB, and 90 dB). But 1 of 14 subjects had tinnitus in the 90 dB environment. In the later stage, 8 subjects were selected from the former 14 subjects to conduct tests with 100 dB,110 dB, and 120 dB to meet the consistency of the noise intensity of the test and the real noise environment in the coal mine.

**Fig. 1:**
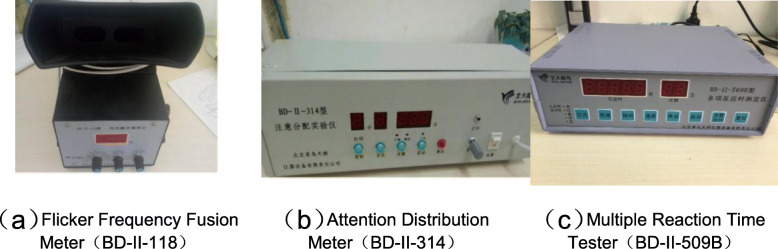
Safety Behavioral Testing System Equipment

In this study: the fatigue level was measured by the flicker fusion critical frequency; the subject’s attention level was measured by their distinguishing different sound and light; the reaction ability was tested by the subject’s reaction time to sound and light. The safety behavioral testing system equipment is shown in Fig. 1.

### Experimental steps

The experiment was divided into two stages, before the experiment and while experimenting.

Preparation before the experiment was as follows: (a) The subjects were told about the test procedure, and trained to use the instrument so that they can operate the instrument, understand the content of the questionnaire and minimize any unnecessary errors; (b) Keep the environmental conditions in advance, including temperature, humidity, and wind speed at a normal level and debugging the equipment. During the whole test period, the subjects should maintain adequate sleep (Not less than 8 h [44]).

The experimental operation involved eight different noise levels. In order to study subjects’ safety behavior ability in each noise level, 14 subjects were divided into seven groups with two subjects in each group (due to the capacity of the experiment devices) in the 50 ~ 90 dB; 8 subjects were divided into 4 groups with two subjects in each group (due to the capacity of the experiment devices) in100 dB, 110 dB, and 120 dB. The test process of one noise level was as follows: First, subjects in a group entered the test environment with a certain noise level, and adapted to the environment for 30 min. Then, their fatigue, attention, and reaction were tested for 30 min and the data were collected. When this group finished, other groups came in the test room one by one and all the data on this noise level were collected. Notably, the numbers of errors made and the reaction time of subjects were recorded synchronously. In general, the whole process for one group of subjects (two people) lasted for 1 h; the actual test time of seven groups of subjects in the 50 ~ 90 dB (altogether 14 people) for each specific noise condition lasted for 7 h in total in a day; the actual test time of four groups of subjects in the 100 ~ 120 dB (altogether 8 people) for each specific noise condition lasted for 4 h in total in a day. The eight different noise conditions were tested on 8 different days, during generally the same period of daytime.

### Statistical analysis methods

This study mainly used two statistical analysis methods: paired-sample t-test and regression analysis. Paired-sample t-test aims to compare the influence difference of two noise levels on human’s safe behavior ability. Specifically, seven experimental groups (60 dB, 70 dB, 80 dB, 90 dB, 100 dB, 110 dB, and 120 dB) were performed paired t-tests respectively with control group (50 dB) and to see if there is a significant difference between the experimental group and the control group, and to find on which noise level workers’ safety behavior may change significantly. To make paired-sample t-test valid, an exploratory analysis of the data is required to determine whether it conforms to a normal distribution before paired-sample t-test. Regression analysis aims to research relevance between independent variable (noise) and dependent variable (attention, reaction, and fatigue). In short, this study firstly studied whether noise affects human safety behavior (attention, reaction, and fatigue), and if so, how does it affect (positively or negatively).

## Result

### Attention

#### Exploratory analysis

As is shown in Table 2, the significant *p*-value of the S-W test of the acousto-optic reaction correct times and that of the Q values were both greater than 0.05 in all 8 noise levels. This presents a normal distribution, and thus paired sample t-test can be performed.

**Table 2:** Exploratory analysis results of attention distribution data

Test items		Shapiro-Wilk		
		Statistics	df	Significant *p*
Control	The number of correct reaction to sound	.984	14	.991
	The number of correct reaction to light	.961	14	.740
	Q value	.966	14	.802
Noise 60 dB	The number of correct reaction to sound	.968	14	.848
	The number of correct reaction to light	.945	14	.479
	Q value	.964	14	.799
Noise 70 dB	The number of correct reaction to sound	.958	14	.688
	The number of correct reaction to light	.977	14	.954
	Q value	.951	14	.525
Noise 80 dB	The number of correct reaction to sound	.925	14	.263
	The number of correct reaction to light	.949	14	.543
	Q value	.944	14	.509
Noise 90 dB	The number of correct reaction to sound	.935	13	.391
	The number of correct reaction to light	.947	13	.560
	Q value	.955	13	.591
Noise 100 dB	The number of correct reaction to sound	.921	8	.437
	The number of correct reaction to light	.979	8	.959
	Q value	.951	8	.525
Noise 110 dB	The number of correct reaction to sound	.900	8	.287
	The number of correct reaction to light	.914	8	.380
	Q value	.928	8	.440
Noise 120 dB	The number of correct reaction to sound	.917	8	.408
	The number of correct reaction to light	.977	8	.946
	Q value	.934	8	.505

#### Sample analysis of t-test

As is shown in Tables 3 and 4, the correct times of the acoustic reactions, the optic reactions and the Q values in the control groups were significantly different from the test values of the control group in 80 dB and above (*P* < 0.05). That is, when the noise is 80 dB or above, the attention level starts to change significantly compared with the control group (50 dB).

**Table 3:** Paired sample t-test results of correct times to sound and light stimuli

	Pairing Difference					t	Degree of Freedom	Significant *P* (Two-tailed)
	Average value (E)	Standard Deviation	Standard Error Mean	95% Confidence Interval for the Difference				
				Lower Limit	Upper Limit			
Sound Control - 60 dB	.785	5.146	1.375	−2.185	3.757	.571	13	.578
Sound Control - 70 dB	1.285	9.769	2.611	−4.355	6.926	.492	13	.631
Sound Control - 80 dB	7.785	11.178	2.987	1.331	14.239	2.606	13	.022
Sound Control - 90 dB	11.461	10.829	3.003	4.917	18.005	3.816	12	.002
Sound Control - 100 dB	4.750	2.764	.977	2.438	7.061	4.860	7	.002
Sound Control - 110 dB	8.125	3.270	1.156	5.390	10.859	7.027	7	.000
Sound Control - 120 dB	11.500	2.828	1.000	9.135	13.864	11.500	7	.000
Light Control - 60 dB	−1.071	2.758	.737	−2.664	.521	−1.453	13	.170
Light Control - 70 dB	1.142	3.483	.930	−.868	3.153	1.228	13	.241
Light Control - 80 dB	4.928	3.852	1.029	2.704	7.152	4.787	13	.000
Light Control - 90 dB	8.153	5.096	1.413	5.074	11.233	5.768	12	.000
Light Control - 100 dB	4.250	1.281	.453	3.178	5.321	9.379	7	.000
Light Control - 110 dB	7.125	2.100	.742	5.369	8.880	9.596	7	.000
Light Control - 120 dB	10.000	2.618	.925	7.810	12.189	10.801	7	.000

**Table 4:** Paired sample t-test results of attention distribution Q value

	Pairing Difference					t	Degree of Freedom	Significant *P* (Two-tailed)
	Average (E)	Standard Deviation	Standard Error Mean	95% Confidence Interval for the Difference				
				Lower Limit	Upper Limit			
Control - 60 dB	−1.061	2.758	.737	−2.664	.521	−1.453	13	.170
Control - 70 dB	1.146	3.483	.930	−.868	3.153	1.228	13	.241
Control - 80 dB	5.029	3.852	1.029	2.704	7.152	3.989	13	.000
Control - 90 dB	6.173	5.096	1.413	5.074	11.233	4.961	12	.000
Control - 100 dB	6.665	5.709	1.595	5.369	8.850	5.134	7	.000
Control - 110 dB	7.023	6.124	1.973	7.449	12.395	5.930	7	.000
Control - 120 dB	8.235	6.802	2.023	8.349	11.044	6.395	7	.000

### Reaction ability

#### Exploratory analysis

Table 5 shows the results of the normality test of the reaction time. From the S-W test in the table, we can see *p* > 0.05. This indicates the reaction time of the acousto-optic is normally distributed on 8 noise levels. Therefore, paired sample t-test can be used to analyze the influence of different noise levels on the reaction time.

**Table 5:** Exploratory analysis results of acoustic and optical reactions

Test items		Shapiro-Wilk		
		Statistics	df	Significant *p*
Control	Light	.954	14	.628
	Sound	.905	14	.132
60 dB	Light	.930	14	.302
	Sound	.902	14	.121
70 dB	Light	.946	14	.499
	Sound	.916	14	.194
80 dB	Light	.946	14	.506
	Sound	.925	14	.262
90 dB	Light	.962	13	.780
	Sound	.929	13	.331
100 dB	Light	.875	8	.169
	Sound	.938	8	.593
110 dB	Light	.918	8	.413
	Sound	.901	8	.297
120 dB	Light	.927	8	.486
	Sound	.937	8	.586

#### Sample analysis of t-test

Table 6 shows that as the external noise level increases, the absolute value of t gradually increases, and t always shows a negative value. It indicates that the test acousto-optic reaction time gradually increases with the increase of noise level. In other words, the greater the noise level is, the more significantly reaction ability decline. When the noise level reaches 70 dB, the reaction time of the subjects to the acoustic stimuli becomes significantly longer; after 80 dB, the reaction time to the optic stimuli becomes significantly longer. These show that the subjects react to optic stimuli better than the acoustic stimuli in the same noise level.

**Table 6:** Paired sample t-test results for acoustic and optic reaction

	Pairing Difference					t	Degree of Freedom	Significant *P* (Two-tailed)
	Average (E)	Standard Deviation	Standard Error Mean	95% Confidence Interval for the Difference				
				Lower Limit	Upper Limit			
Sound Control - 60 dB	−.009	.045	.012	−.035	.017	−.765	13	.458
Sound Control - 70 dB	−.044	.054	.014	−.075	−.012	−3.052	13	.009
Sound Control - 80 dB	−.077	.088	.023	−.128	−.026	−3.266	13	.006
Sound Control - 90 dB	−.136	.072	.020	−.180	−.092	−6.785	12	.000
Sound Control - 100 dB	−.106	.026	.009	−.129	−.084	−11.196	7	.000
Sound Control - 110 dB	−.139	.030	.010	−.165	−.113	−12.914	7	.000
Sound Control - 120 dB	−.198	.022	.007	−.216	−.179	−25.282	7	.000
Light Control - 60 dB	−.001	.010	.003	−.007	.004	−.530	13	.605
Light Control - 70 dB	−.008	.014	.004	−.016	.001	−2.337	13	.056
Light Control - 80 dB	−.060	.039	.010	−.082	−.037	−5.778	13	.000
Light Control - 90 dB	−.093	.035	.010	−.114	−.072	−9.524	12	.000
Light Control - 100 dB	−.087	.027	.009	−.109	−.065	−9.220	7	.000
Light Control - 110 dB	−.134	.029	.010	−.158	−.110	−13.255	7	.000
Light Control - 120 dB	−.181	.037	.013	−.212	−.150	−13.957	7	.000

### Fatigue

#### Exploratory analysis

S-W analysis results show that *p* > 0.05. It indicates that the subjects’ flicker fusion critical frequency is in normal distribution on 8 noise levels. The specific analysis results of S-W are shown in Table 7.

**Table 7:** Exploratory analysis results of noise group flicker fusion frequency

Noise level	Shapiro-Wilk		
	Statistics	df	Significant *p*
Control	.959	14	.701
60 dB	.977	14	.952
70 dB	.956	14	.654
80 dB	.980	14	.977
90 dB	.958	13	.730
100 dB	.859	8	.118
110 dB	.853	8	.103
120 dB	.897	8	.273

#### Sample analysis of t-test

Table 8 shows that the t value of paired sample test increases with the increase of the noise. Flicker fusion critical frequency decreases as the noise increases. It can be concluded that the worker’s fatigue increases with the increase of noise. Table 8 shows that there is a significant difference between the control group and the 70 dB group and above 70 dB groups. In other words, the external noise has a significant impact on fatigue from 70 dB.

**Table 8:** Paired sample t-test results of flicker fusion frequency

	Pairing Difference					t	Degree of Freedom	Significant *P* (Two-tailed)
	Average (E)	Standard Deviation	Standard Error Mean	95% Confidence Interval for the Difference				
				Lower Limit	Upper Limit			
Control - 60 dB	.014	.751	.200	−.419	.448	.071	13	.944
Control - 70 dB	.407	.628	.167	.044	.769	2.425	13	.031
Control - 80 dB	1.657	.873	.233	1.152	2.161	7.100	13	.000
Control - 90 dB	2.107	.864	.239	1.585	2.630	8.790	12	.000
Control - 100 dB	3.050	.728	.257	2.440	3.659	11.834	7	.000
Control - 110 dB	3.450	.621	.219	2.930	3.969	15.712	7	.000
Control - 120 dB	4.125	.686	.242	3.551	4.698	17.006	7	.000

*In Table 2, 3, 4, 5, 6, 7 and 8: In order to facilitate observation, the data analysis results are marked. If Significant *P* (*p*) ≥ 0.05, gray mark is used, and Significant *P* (*p*) < 0.05, orange mark is used

### Prediction of the impact of noise levels on workers’ safety working ability

As can be seen from the above analysis, noise has a significant influence on fatigue, reaction, and attention. In order to find out the relationship between safety behavioral indicators and noise, we took noise level as independent variable and behavior indicators as the dependent variable. The experimental data was subjected to regression analysis. The regression process selects linear, logarithmic, quadratic, power function, and exponential function. The best fit models were selected based on R^2^. When R^2^ is greater than 0.9, the data fitting effect becomes better.

**Fig. 2:**
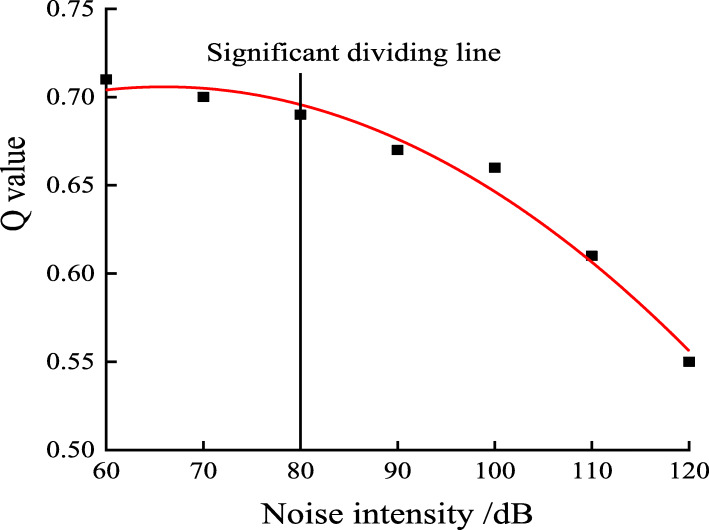
Relationship between attention distribution Q value and noise levels

**Fig. 3:**
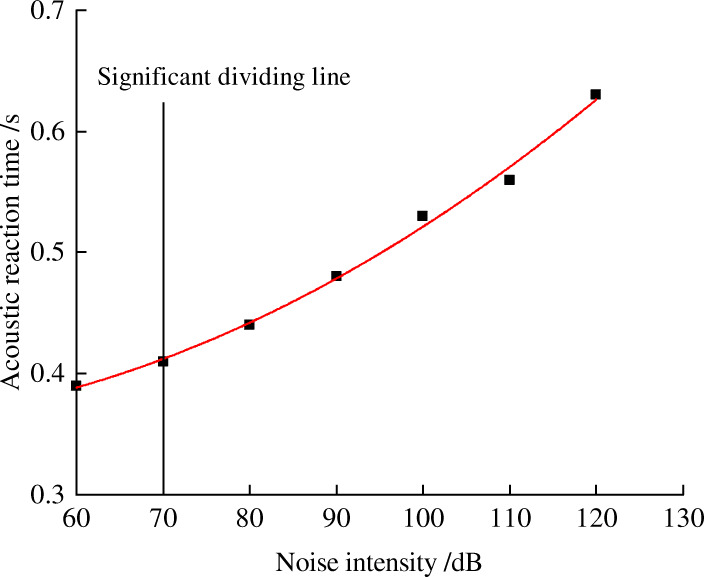
Relationship between acoustic reaction time and noise levels

**Fig. 4:**
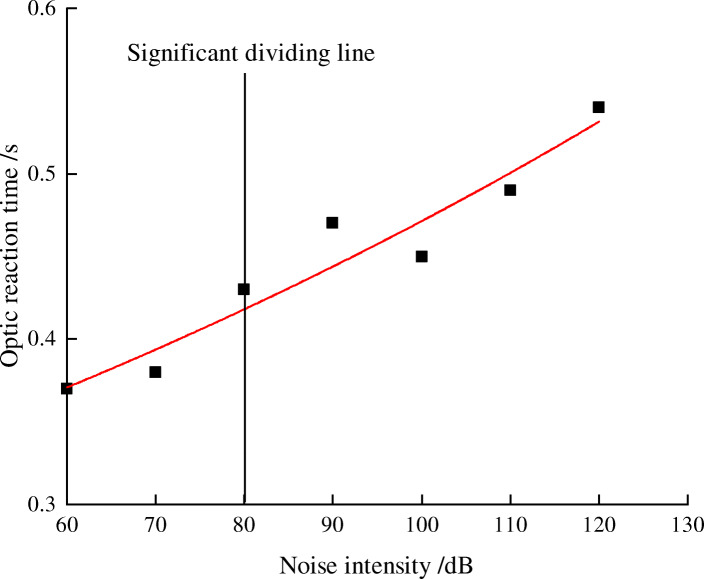
Relationship between optic reaction time and noise levels

**Fig. 5:**
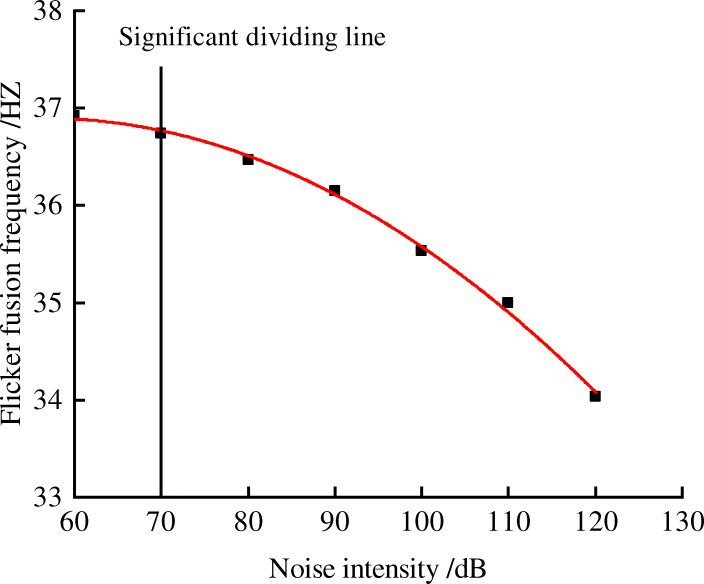
Relationship between flicker fusion frequency and noise levels

Figures 2, 3, 4 and 5 shows the trend between noise and behavior indicators. Noise is negatively correlated with the attention and reaction, and it is positively correlated with fatigue. When environmental noise level exceeds 70 ~ 80 dB, noise has a significant effect on the subjects’ attention, reaction, and fatigue.

It can be seen from Fig. 2 that when the noise level is between 60 and 80 dB, Q value decreases slowly with the increase of noise level. When noise level is greater than 80 dB, the Q value decreases sharply. When the noise level reaches 120 dB, the subject’s Q value is 0.55, which is close to the distraction allocation. If the noise level continues to increase, the attention of the subjects will be seriously affected.

As shown in Figs. 3 and 4, with the increase of noise levels, the acousto-optic reaction time increases correspondingly, the comparison between the reaction time to acoustic stimuli and light stimuli shows that the reaction time to the acoustic stimuli varies from 0.40s to 0.63 s and the reaction time to the optic stimuli varies from 0.37 s to 0.55 s. The reaction time to acoustic stimuli will be longer. Acoustic reaction time becomes significant when the noise is 70 dB, while optic reaction time is 80 dB. It shows that subjects are more sensitive to optic stimuli than acoustic stimuli in noisy environment.

It can be seen from Fig. 5 that when noise level is lower than 70 dB, the change of flash fusion frequency value is minor. When noise level reaches 70 dB or more, flash fusion frequency decreases greatly. It shows that different levels of noise have different effects on the fatigue degree. The greater noise level is, the more fatigue the subjects are.

From the analysis of fitting effect as is seen in Table 9, exponential function and quadratic function are most suitable for the modeling of the data in this study. The derivative value of the function indicates the speed of change of the behavior indicators. According to the properties of exponential functions and quadratic functions, the absolute value of derivative of the two functional models continue to increase. Therefore, the greater the noise level is, the faster attention, reaction and fatigue will change, so workers are more prone to accidents in high noise environment.

**Table 9:** Fitting equation of behavior indicators and noise levels

Behavior index	Regression fitting equation	R^2^
Q value	y = −0.0051 × ^2^ + 0.0163x + 0.6929	0.9813
Reaction time of sound	y = 0.00003 × ^2^–0.0014x + 0.3701	0.9966
Reaction time of light	y = 0.2587e^0.006x^	0.9241
Flicker fusion frequency	y = −0.0007 × ^2^ + 0.079x + 34.668	0.9969

*In formula: x is the noise level, R^2^ is the correlation coefficient, indicating the accuracy of the fitting

## Discussion

Unlike most of the previous studies [6, 9–12, 26], which studied the occupational harm of noise on human, this study focused on the influence of different noise levels on miners’ safety behavior in underground coal mines and conducted a simulation quantitative experiment of 93 people/hours. Results of this study show that high noise environment significantly affect fatigue, attention, and reaction. Significance analyses reveal that fatigue is the most sensitive to the change of noise and displays a significant change when the noise is above 70 dB. The sensitiveness of reaction and attention are followed by that of fatigue and displays a significant change when the noise is above 80 dB. In the noise environment, the sensitivity of optic stimuli is more obvious than that of acoustic stimuli. In this sense, optic stimuli can be used to improve safety systems in noisy environment. Regression analysis results show that noise is negatively related to attention and reaction, and positively related to fatigue.

At the same time, this study has the following shortcomings: (a) The number of subjects in this experiment is small, and the age of the subjects is different from that of the actual miners. (b) Due to the limitation of our experimental conditions, the experiment did not include the influence of the time duration of the noise exposure. As a fundamental study in the field of coal mine noise, this paper mainly aims to measure the behavioral indicators of subjects affected by noise environment. To address the limitations of the study, we will conduct an in-depth study on the impact of the noise environment on the safety behavior of miners by expanding the sample size and measuring noise duration.

Similar with the previous studies [35–37], this study also found that the safety behavior ability of miners in a high noise environment is significantly lower than that in a low noise environment. However, we found a noise level of 70 ~ 80 dB starts to affect the safety behavior ability while other researches [35–37] concluded that the specific noise level that changes significantly is different (85 ~ 95 dB). The reason for the difference between previous studies and this study may be different sources of noise, different safety behavior indicators, different subjects and different interests (the previous studies focused on physical health, while this study focused on safety behavior). But certain reasons need to be studied in-depth. There are suggested pathways linking long-term exposure to noise environment and human unsafe behavior. In a study by Deng [33], noise affected the physiology and psychology, and then affected human behavior, increasing the probability of safety accidents. Specifically, in the high-noise environment, such effects are manifested as the distraction of attention, the decrease of auditory ability. They lead to auditory and systemic fatigue. In this state, due to the development of protective inhibition, the activity of cerebral cortex cells decreases. Accordingly, the conditioned reflex activity is affected, the probability of mis operation increases, and the probability of accident increases.

## Conclusion

This paper selected three safety behavior indicators: attention, reaction, and fatigue, and studied how coal mine noise effects these safe working abilities. The results were shown as follows:Noise can affect the attention, reaction, and fatigue of miners. When the environmental noise is 80 dB or above, the attention begins to change significantly compared with an environment without noise (50 dB). When the noise is 70 dB or above, fatigue level begins to show a significant change compared with an environment without noise (50 dB). Notably, we found the sensitivity of optic stimuli is more obvious than that of acoustic stimuli: the reaction time to acoustic starts to be statistically significant from 70 dB while the reaction time to optic starts to be statistically significant from 80 dB. In this sense, optic stimuli can be used to improve safety systems in noisy environment. Results of regression analysis show that attention and reaction is negatively related to noise levels, while fatigue is positively related to noise levels. Taking noise as the independent variable, attention (Q value), fatigue (flash fusion frequency), and acoustic reaction time is best fitted by the mathematical model of quadratic function. Optic reaction time is best fitted by exponential function. And Figures 2, 3, 4 and 5 shows that compared with the no noise (50 dB), the greater the noise level increases, the more significantly the subjects’ attention, reaction, and fatigue change. It infers that workers are safer in a low noise environment. It is recommended that the noise level in working place is controlled within 70 ~ 80 dB or below. This way, the inclination of accidents will decrease.

## Data Availability

The datasets used and/or analysed during the current study are available from the corresponding author on reasonable request.
